# Nutritional Benefits from Fatty Acids in Organic and Grass-Fed Beef

**DOI:** 10.3390/foods11050646

**Published:** 2022-02-23

**Authors:** Hannah Davis, Amelia Magistrali, Gillian Butler, Sokratis Stergiadis

**Affiliations:** 1 School of Natural and Environmental Science, Newcastle University, Newcastle-upon-Tyne NE1 7RU, UK; amelia.magistrali@newcastle.ac.uk (A.M.); gillian.butler@ncl.ac.uk (G.B.); 2 Department of Animal Sciences, School of Agriculture, Policy and Development, University of Reading, Reading RG6 6EU, UK

**Keywords:** ruminant nutrition, fatty acids, nutritional quality, organic, pasture-fed, conventional

## Abstract

Livestock production is under increasing scrutiny as a component of the food supply chain with a large impact on greenhouse gas emissions. Amidst growing calls to reduce industrial ruminant production, there is room to consider differences in meat quality and nutritional benefits of organic and/or pasture-based management systems. Access to forage, whether fresh or conserved, is a key influencing factor for meat fatty acid profile, and there is increasing evidence that pasture access is particularly beneficial for meat’s nutritional quality. These composition differences ultimately impact nutrient supply to consumers of conventional, organic and grass-fed meat. For this review, predicted fatty acid supply from three consumption scenarios were modelled: i. average UK population National Diet and Nutrition Survey (NDNS) (<128 g/week) red meat consumption, ii. red meat consumption suggested by the UK National Health Service (NHS) (<490 g/week) and iii. red meat consumption suggested by the Eat Lancet Report (<98 g/week). The results indicate average consumers would receive more of the beneficial fatty acids for human health (especially the essential omega-3, alpha-linolenic acid) from pasture-fed beef, produced either organically or conventionally.

## 1. Introduction

Consumer awareness of the environmental impacts of ruminant production has grown over the past two decades [1]. Although global demand for meat is predicted to increase by 1.3% per year up to 2050, this is a lower growth rate than previous periods, likely affected by slower population growth and modest expected increases in per capita meat consumption in the Global North [2]. Greenhouse gas (GHG) emissions from livestock supply chains are estimated to represent 14.5% of all ‘human-induced’ emissions, with beef cattle producing 3 Gt CO_2_ equivalents [3,4,5]. Research rhetoric focused on mitigating the impacts of climate change emphasise that moving to plant-based diets will reduce GHG emissions. This appears to be led by the International Panel on Climate Change (IPCC) [6] and supported by interest-group-funded research, such as the Lancet commissioned report on ‘healthy diets from sustainable food systems’ [7]. Vegetarianism and veganism are rising in the UK and many other countries in the Global North, not only in food sales but also in participation in campaigns to reduce consumption of meat and other animal food products [8]. Notably, in behaviour analysis of UK participants in vegan and meat-reduction campaigns, red meat is the most likely product for planned reductions and abstentions [8]. Limitations to red meat consumption have also been recommended by nutritional guidelines based on epidemiological meta-analyses showing ‘probable’ evidence that increased red meat consumption increases the risk of colorectal cancer and premature death [9].

Though many interest groups point to plant-based diets to combat the environmental consequences of industrial meat production, organic and grass-fed meat are realistic but lesser-discussed alternatives. Consumer demand for organic meat has slowed in recent years, from growth periods centred around 2008 [10]. Although the overall value of organic beef sales decreased in the US between the 2014 and 2019 organic agriculture census [11], the number of organically reared cattle in the UK has remained stable from 2019–2020 [10]. Even as organic makes up a minor proportion of total livestock production (3.2% of the total UK beef cattle population is organic [10]), organic animal agriculture is still often grouped with all livestock production in assessing GHG emissions [3,4]. A 2020 *Nature* article analysing the external climate costs of food determined that external GHG costs are highest for both conventional and organic animal-based products (including eggs, poultry, ruminants and pork) compared to milk and plant-based foods [12]. Despite this grouping of all animal agriculture as ecologically unsustainable, there are clearly differences in environmental impact between production systems [5,13,14], especially when considering impacts beyond emissions, including biodiversity [13,15], soil health [13,16] and energy/land use [13].

While a shift to more plant-based diets seems inevitable, there is also evidence that animal products will continue to be staples in many diets worldwide [17], and ruminant meat is incredibly nutrient dense, a good source of high-quality protein, with beneficial fatty acids and important micronutrients [18,19]. As already noted, global consumption of meat products is expected to grow, and there is clear evidence that while meat consumption initially increases with income (as in China and Brazil), there is a point at which high income actually contributes to lower meat consumption (evident in Denmark and France) [20]. Beyond economics, there are also many political, institutional, cultural and social factors affecting consumption habits in different countries [17,21,22], but the fact remains that red meat is an important source of nutrition [18,19]. As the globe grapples with the necessity for sustainable food production, considering the impact of management practices on nutritional quality is increasingly valuable.

This paper aims to review the current literature and explore whether organic and/or pasture-reared beef confers nutritional benefits over conventionally produced beef. 

## 2. Beef Production Principles and Standards

Standards for ruminant meat production vary regionally and within different countries. These range from governmental food standards and animal welfare legislation to standard certification bodies, which include, but are not limited to, organic standards. These standards are regulated and often offer the farmer a premium above the average market price. However, there are also values that farmers may hold themselves accountable to, such as low-input, sustainable, regenerative, agroecological or conservation. These principles are not regulated but may give farmers a premium depending on their market. As the details of specific certification schemes and management principles vary greatly globally, this study will focus on describing different management standards that influence beef’s nutritional properties, using examples of standards from the UK and the European Union.

### 2.1. Concentrate vs. Forage-Based Diets

Traditionally, the ruminant diet has come from grazing pasture, as their digestive systems have evolved to use forage. Modern industrialised meat production (defined by higher stocking rates and breeding for rapid growth) requires higher proportions of concentrate feed, including increased protein consumption, to allow for quick turnover to slaughter-weight of prime animals. However, ruminant digestion remains most suited to forage-based diets [23] and the nutritional quality of meat is impacted by animal nutrition [19,24,25]. This push-pull of a forage-based diet vs. increased productivity highlights key differences between ruminant livestock standards.

Baseline standards for animal welfare in the UK include the government welfare codes and the Royal Society for the Prevention of Cruelty to Animals (RSPCA), which both emphasise that livestock must be fed a diet appropriate to their species, which satisfies nutritional needs and maintains good health [26,27]. The provisions of these kinds of standards do not dictate a certain proportion of diet from forage, but suggest that animals should spend time outdoors with access to pasture provided they have adequate food, water and shelter. The ‘Five Freedoms’ of welfare standards in the UK include ‘freedom to express normal behaviour’, which is further dictated by stocking densities (10–12 animals/acre for cattle), minimum indoor lying areas and access to pasture that does not cause harm [26]. Non-organic standards for livestock typically do not dictate a minimum outdoor access requirement. For example, the UK Red Tractor certification focuses on providing comfortable, hygienic and sufficient housing/shelter as well as a suitable diet, but does not specify outdoor access as essential or a minimum requirement for forage-based feed [28]. 

Under organic standards, the importance of grazing and forage intake (when pasture is not available) is made more explicit, as well as specific regulations for feed sourcing. EU regulations for organic livestock management require that animals have access to open air or grazing areas whenever possible and only consume organically produced feed (concentrate or forage) [29]. In some countries, as in the UK, organic standards are even more specific, stating a minimum of at least 60% of ruminant diets must be fresh/dried fodder, roughage or silage [30,31]. 

An additional category of livestock management standard that has become more common over the past decade focuses specifically on access to pasture and forage feeding. Based on the requirements for certification, farmers meeting these standards may or may not be organic and/or qualify for other quality assurance schemes. The Pasture for Life Certification Mark of the Pasture-Fed Livestock Association (PFLA) (in the UK) specifically emphasises management based exclusively on pasture, explicitly stating that zero-grazing systems (feeding cut grass to housed animals) are prohibited and that livestock must be maintained outdoors on pasture [32]. Specifically, the PFLA requires that livestock must be maintained on rotation pastures, permanent pasture, fields of forage crops or on the unbroken ground at all times except for over-winter periods, under conditions leading to soil damage and/or risk animal welfare or following community/national biosecurity requirements [32]. Many PFLA farmers may also be certified organic, but the scheme does not specify that forage must be certified organic, which means that non-organic farmers can also qualify based on their pasture and forage management practices. 

### 2.2. Definitions for Feeding Systems in the Present Paper

For the purposes of the analysis in this review, which covers studies in countries with varying standards, the different feeding systems will be categorised as one of the following:

- Intensive (INT) refers to conventional management with extremely limited access to preserved forage (excluding cereal straw, which is often the main ‘forage’ in the diet), animals are fed ad-lib concentrate feeds to appetite (typically composed of soya, grains and molasses) and kept exclusively indoors or in feedlots.
- Conventional (CON) refers to non-organic management adhering to country-specific animal welfare standards, but otherwise not restricted by regulations for minimum proportion of forage in diet (majority concentrate feeding, but typically ~30% roughage or forage- could be mixed crop, grass or maize) and/or outdoor access.
- Organic (ORG) refers to management adhering to country-specific standards, including organically certified feed (concentrate, silage or hay and pasture). Any other feed requirements regarding forage consumption are specified based on source material.
- Pasture-based (PB) refers to management centred on access to pasture/forage, including: 100% forage feeding with most consumed by grazing. Unless otherwise stated, PB is used to discuss cattle that have been reared and finished on pasture/forage.

When referencing specific studies throughout this review, the terminology and feed system described in each paper will be used, which means that in some cases ‘grass-fed’ is defined by the referenced study, and is not necessarily 100% pasture-based (PB) as outlined above. There is a wide diversity of finishing systems within the beef industry that impact lipid content and fatty acid profile, for example, pasture-reared but intensively finished (often used in suckler systems, with varying finishing times) [33,34]. Additionally, the composition of preserved forage can also impact beef fat composition, mainly in comparing maize vs. grass silage [35]. We recognise that additional factors related to feeding systems impact fatty acid profile, but for the purposes of this paper have focused our attention on the definitions specified in this section. 

## 3. Beef Quality

The quality of animal-source products, as perceived by consumers, is evolving and includes several drivers. Consumers are not only interested in flavour, shape and tenderness but also origin, potential impact on health, brand and antibiotic use [36,37]. Prache et al. [9] define seven animal-source product attributes: (i) Sensory, (ii) Nutritional, (iii) Image, (iv) Convenience, (v) Safety, (vi) Technological, (vii) Commercial. In this review, quality focuses on nutrition-relevant fatty acids (nutritional), in the context of meeting nutrition guidelines and potential impacts on human health (and safety).

According to the McCance and Widdowson’s composition of foods integrated dataset, an indicative beef nutrient composition (average from 23 different cuts in the database) would be approximately 68.5% water, 20.9% protein and 9.5% fat; translating to 58.6% water, 29.4% protein and 10.6% fat when these cuts are cooked [38]. Meat is generally considered a key source of protein, especially essential amino acids, which cannot be synthesised in the human body [19,25]. Global figures indicate that all meat provides 20–40% of protein intake in the human diet, and in most countries in the Global North, average protein consumption provides more than the minimum requirement needed for good health [25]. Red meat, in particular, contains high-value protein, including all eight essential amino acids required by adults and all nine amino acids required by children [19]. Current evidence suggests protein quantity is very similar between organic and conventionally produced meat [39,40]; however, the fat content of meat is much more variable than protein, as there is a stronger influence of animal type and production factors. Nutritionally, fat provides rich sources of energy, but also essential vitamins and fatty acids and contributes to palatability and flavour [25].

In meat, there are three fat categories: (i) inter-muscular, occurring between the muscles; (ii) intra-muscular (IM) fat, commonly known as ‘marbling’; (iii) subcutaneous (SC) fat, the deposition layer between skin and muscle [25]. This paper focuses on the most commonly consumed fat, IM fat. The amount of fat and the fatty acid profile in IM fat is driven by diet [41], with clear differences based on breeds [41,42], slaughter age [41,43], nutrition [41] and between muscle types [40,44]. As marbling is affected by all these factors, including country-specific consumer preferences, IM fat content in beef varies, with high percentages in the US (up to 11%) and Japan (20%) and lower amounts in France (up to 6%) [45]. A review of intramuscular fat content and properties by Park et al. [41] presents a range in IM fat from 1.9% in Brahman cattle in the Philippines [46] to 37.8% in Japanese Wagyu beef [47]. Higher IM fat content is associated with high concentrate diets compared with low concentrate diets [41,43], including higher IM fat content in feedlot finished compared with grass-finished beef [48]. 

## 4. Fatty Acids

Dietary fat is mostly (99%) comprised of acyl-glycerols and phospholipids and all fat from food consumed will have varying amounts and types of fatty acids (FAs). Fatty acids are carboxylic acids classified by the length of their carbon chains, whether they have double bonds and the configuration of the hydrogen atom [49]. The main categories of fatty acids in beef are: saturated fatty acids (SFA, approximately 46% of total raw, lean IM FA) with no double bonds, monounsaturated fatty acids (MUFA, approximately 46% of total raw, lean IM FA) with one double bond and polyunsaturated fatty acids (PUFA, approximately 7% of total raw, lean IM FA) with two or more double bonds [38]. Early dietary guidance grouped and researched FAs of the same class (e.g., SFAs), whereas more recent research has studied the impact of individual FAs (e.g., linoleic acid) for their effect on human health. While this approach is reasonable in a research context, explaining the health impacts of individual fatty acids to consumers would be a confusing approach to nutrition, and instead well-rounded dietary advice from FAs is recommended [50]. Many FAs are recognised as having positive and/or negative health outcomes for consumers and in some cases, the relative concentrations or ratios of one to another may be of more importance than absolute intakes [51].

### 4.1. Saturated Fatty Acids

Saturated FAs have historically all been considered undesirable in the human diet [52]. The main SFAs in ruminant meat products are: myristic (C14:0), palmitic (C16:0) and stearic (C18:0) acids. Some SFAs (lauric, myristic and palmitic) have been shown to have cholesterol-increasing properties, which are an indicator of coronary heart disease (CHD) risk [53]. Generally, elevated low-density lipoprotein cholesterol (LDL-C) is associated with a higher risk of heart/artery disease than high-density lipoprotein cholesterol (HDL-C), which is protective [54,55]. The links and mechanisms between SFA, cholesterol and CHD are complicated and often conflicting, as individual SFA have been linked to positive, neutral and negative effects on heart disease [53]. Forouhi et al. [56] found even chain SFAs (C14:0, C16:0 and C18:0) were positively associated, while odd chain SFAs (C15:0 and C17:0) (of rumen origin and found in minor concentrations) were inversely associated with the incidence of Type 2 diabetes. Khaw et al. [57] also reported even chain SFAs were positively associated with CHD risk. It is clear not all SFAs affect human health uniformly, suggesting that further subgrouping and identifying specific functions of individual FAs can help identify risk factors for human health.

### 4.2. Monounsaturated Fatty Acids

Monounsaturated FAs have one double bond somewhere along the carbon chain with the ‘remaining’ hydrogen in either the cis or trans configuration, as demonstrated in oleic (OA, c9 C18:1) and vaccenic (VA, t11 C18:1) acids, respectively. Oleic acid is the most abundant MUFA in beef and is commonly found in animal fats, olive oil, nuts and avocados, while VA is solely found in ruminant fats [58,59]. Meta-analyses have shown that replacing SFAs with cis-MUFAs can reduce LDL and increase HDL (small effect size) [53,60], although this could be due to the reduction in SFAs rather than an increase in OA. Another meta-analysis, which did not distinguish between cis- and trans-MUFAs, found no difference in health outcomes between the FAs consumed [61] and Vafeidou et al. [62] reported a reduced risk of cardiovascular disease when SFAs are replaced with MUFAs, although this mechanism is not yet fully understood. If studies reduce specific FAs and replace them with other FAs, is it the reduction in SFA content or the replacement that is responsible? The answer to this question is still unclear and since the proportion of all FAs and FA groups are interlinked (all expressed as a proportion of their total), considering them in isolation does not always provide clear guidance. 

The main trans fatty acid (TFA) in beef is the MUFA VA (t11 C18:1), whereas the predominant trans FA in industrially hardened, hydrogenated oils is the MUFA elaidic acid (t9 C18:1), an important distinction because their metabolism is different [63]. There are many health concerns surrounding TFAs, including associations with increased risk of CHD, obesity and insulin resistance [64]. Vaccenic acid is metabolised to rumenic acid (c9t11 CLA; CLA9) (discussed below), which is beneficial for human health, in the adipose tissue of both animals and humans, whereas elaidic acid has been closely linked to CHD, steatohepatitis and obesity [65]. The naturally occurring TFAs found in meat may not be harmful [66] but due to the challenges of isolating TFAs and examining their direct effect on human health, the UK recommendation is no more than 2% of dietary energy intake should come from TFAs [67]; however, the evidence does not point towards ruminant-derived TFAs (VA) negatively impacting human health, and in particular, health benefits have been reported as a result of VA consumption [58].

### 4.3. Polyunsaturated Fatty Acids

Polyunsaturated fatty acid (PUFA) research has become very popular in human nutrition. PUFAs are categorised as having more than one double bond and most are classified into two main groups: omega-3 (*n*-3) FAs have a double bond between the third and fourth carbon from the end methyl group, and omega-6 (*n*-6) FAs have a double bond between the sixth and seventh carbon from the end methyl group [68]. Many FAs can be metabolised and synthesised by the human body, but there are two main essential PUFAs that must come from the diet: *n*-3 α-linolenic acid (ALA) and *n*-6 linoleic acid (LA) [50,68]. Interventional and observational studies demonstrate that replacing SFAs in the diet with PUFAs significantly reduces cardiovascular disease (CVD) risk [52,62,69]. 

#### 4.3.1. Omega-3 Fatty Acids

The main omega-3 PUFA is the essential ALA, which metabolises to the long-chain FAs (LCFAs with chains of >18 C) eicosapentaenoic acid (EPA, C20:5), docosapentaenoic acid (DPA, C22:5) and docosahexaenoic acid (DHA, C22:6) (*n*-3 FAs with more than 20 carbons). DHA is an important part of all cell and organelle membranes and is found in the brain and retina [70]. LCFAs are found in fish and fish oil products and in much smaller quantities in meat, eggs and dairy. Research suggests that conversion of ALA to EPA, DPA and DHA is very limited (1–8% ALA converted to EPA depending on the method of analysis and LA concentration in diet) [51,71,72], signifying the importance of getting these nutrients directly from the diet [73]. In the Global North, the typical consumption of ALA and LCFAs is below recommended levels [74]. Many countries recommend 500 mg LCFA per day, yet average populations often do not consume half of that recommendation, as is the case in seven European countries (~239 mg) [75], including Germany (~160 mg) [76,77]. These FAs are vital for foetal development [78], healthy aging and neuro-development and neuro-degeneration [79], controlling inflammation, FA metabolism and may have a protective role against CVD [80] and prevent some cancers [81]. In recent history, there has been a decline in *n*-3 consumption (generally, eating less fish, pasture raised ruminant produce and nuts and seeds) in the Global North [82], yet this research highlights the benefits of consuming a diet rich in *n*-3 FAs overall.

#### 4.3.2. Omega-6 Fatty Acids

The most prevalent *n*-6 in animal and human diets is the essential linoleic acid (LA), found in plants and seeds and metabolised to the long-chain FA arachidonic acid (AA) (abundant in muscle and specifically ruminant products) [50]. LA is important for skin barrier function [83], whilst AA has an important role in brain development and function [84] and synthesis of eicosanoids [80]. These FAs are generally proinflammatory [85], which helps to defend against pathogens, but if there is a loss in the regulation of inflammation, disease can occur [80]. In modern diets in the Global North, consumption of *n*-6 has risen sharply over the previous 150 years with the increased use of vegetable oils and cereal grains (along with a decrease in fresh vegetables) [86]. This over-consumption of *n*-6 and under-consumption of *n*-3 has potentially led to inflammatory processes, linked to an increase in diabetes, obesity and atherosclerosis [87,88]; however, replacing SFAs with LA has been shown to lower blood cholesterol and LDL [53], suggesting that LA could lower CVD when replacing SFAs. Adding to the complications of defining health impacts of omega-6 FAs, Chowdhury et al. [61] found no association that *n*-6 intake affected CHD risk. Despite this, some eicosanoids promote tumour growth, which is speculated to be in response to increased AA levels, but the conversion of LA to AA is extremely low (around 0.5%) [89]. Ultimately, LA is an essential FA and current research suggests that any increase in consumption of PUFAs is advantageous and reducing overall *n*-6 consumption is not advised.

#### 4.3.3. Omega-6:Omega-3 Ratios

Linoleic acid and alpha-linolenic acid share a complement and competitive metabolic pathway [90], with reactions mediated by the same enzymes [91]. Due to this competition for metabolism enzymes, the ratio of *n*-6:*n*-3 (and specifically LA:ALA) is important. Notably, LA metabolising to AA tends to take priority over the ALA to EPA, DPA and DHA pathway [92], again, making the relative ratio an important aspect of animal and human nutrition and health, since high LA intakes dominate the shared enzymes responsible for *n*-3 LCFA synthesis.

Typical diets in the Global North have an *n*-6:*n*-3 ratio ranging from 7:1 to 20:1, far from the 1:1, which is thought to have been the norm during human evolution [50,93]. Historically, *n*-3 came from fish, meat and dairy (reared on pasture), eggs, leafy green vegetables, nuts and berries, but their consumption has decreased whilst *n*-6 consumption has increased [86], as described in the previous section. There is increasing evidence this excess LA consumption and increase in dietary *n*-6:*n*-3 ratio has contributed to the rise in obesity, atherosclerosis and diabetes [85,87,88]. The imbalance in this ratio towards *n*-6 is also highly proinflammatory and prothrombotic [88]; therefore, working towards an *n*-6:*n*-3 ratio between 1:1 up to 4:1, over the whole diet, is considered beneficial for human health [86]; however, much of this evidence seems to be based on pathways rather than robust dietary intervention trials or long-term health studies. At least for now, the evidence does not suggest that *n*-6 consumption should decrease; however, raising consumers’ dietary *n*-3 would increase total PUFA intake (preferably by replacing SFAs) and decrease the *n*-6:*n*-3 ratio.

#### 4.3.4. CLA

A group of linoleic acid isomers is conjugated (CLAs) (having double bonds on adjacent carbon atoms), the most abundant being CLA9, which is mainly found in ruminant milk and meat, together making around 90% of human intake [94]. Technically, although they contain trans- bonds, they also have a cis- double bond and are mostly omitted from the trans-fat category [74]. CLA9 has attracted attention due to identified anti-cancer properties and health benefits associated with the immune system and cardiovascular health [95,96] as well as the potential to reduce adiposity [97]. Much of the research examines pathways and mechanisms or discusses studies based on animal models (predominantly mice); however, Dilzer and Park [98] reviewed studies involving humans and Yang et al. [99] reviewed health and mechanistic studies, both concluding there is evidence of health benefits from CLA, but the observed effects are larger in mice and more research is needed to understand the dose effect in humans. 

## 5. Origins of Fatty Acids in Meat

The ruminant diet is predominantly (up to 70% of dry matter) carbohydrates (fibre, starch and some sugar), which is broken down in the rumen to simple sugars and converted to pyruvate [100]. Pyruvate is then converted to the volatile fatty acids (VFAs) acetate, propionate and butyrate (and carbon dioxide and methane) in the rumen [100]. The relative proportion of the VFAs is influenced by the animal’s diet and time elapsed since the previous meal [100]. For example, fibrous forages create more acetate, whereas more digestible forages increase the concentration of propionate [100]; however, when concentrates make up a high proportion of the diet, as with intensive beef, propionate increases at the expense of acetate [100]. These energy-providing fermentation end-products are essential for a healthy rumen; acetate and butyrate are then used for de novo fat synthesis and propionate is used for glucose [23]. 

Additionally, lipids are a small but important component of the ruminant diet (up to 8% of dry matter). The FA profile of forages (such as grasses and clovers) are dominated by ALA (~62% of the total), LA (~20%) and palmitic acid (C16:0) (~17%), which combine to ~93% of the profile, though the relative proportion in different forages is variable [101,102]. In contrast, grain lipids (such as cereals and oilseeds in concentrate feeds) have a higher proportion of LA (~58%) compared to ALA (~4%) and palmitic acid (~20%) [103,104]. In one study using Aberdeen Angus steers slaughtered at 14 months, meat LA concentration was higher in the cattle fed concentrate compared to grass silage (119 vs. 46.6 mg/100 g IM), whilst the opposite was true for ALA content (4.0 vs. 20.6 mg/100 g IM) [42]. This, in part, is due to the different fatty acids entering the rumen but also rumen retention. Concentrate-based LA-rich diets contain smaller particles that pass through the rumen faster than forage diets, decreasing the time spent in the rumen and limiting biohydrogenation resulting in more LA leaving the rumen [43]. The uptake and metabolism of dietary FAs into FAs in the muscle tissue have been in detailed illustrated in previous work [105]. Both carbohydrates and lipid sources, influenced by feeds in the diet, will have an impact on the FA profile of meat, directly via the FAs supplied and indirectly from rumen fermentation and VFAs produced [106]. This indicates that ruminant nutrition can be a tool to enhance meat FA profiles. 

The rumen bacteria and protozoa hydrolyse esterified fat into unsaturated free fatty acids, phospholipids and glycolipids (and other organic compounds) [107]. Then, most unsaturated FAs are bio-hydrogenated, resulting in mostly saturated free FAs leaving the rumen, ultimately predominantly palmitic (C16:0) and stearic acid (C18:0) [108]. Despite this, intermediate pathways give rise to many, more minor, FAs [109]. The enzyme stearoyl Co-A desaturase in adipose tissue has been well studied for its action in converting VA to CLA9 and oleic acid (c9 C18:1) from stearic acid (C18:0) [37,43]. There is some evidence that the essential fatty acids are favourably stored in the IM fat over SC, potentially due to their metabolic roles [42]. This further emphasises the nutritional variability potential of beef fat. 

## 6. Effect of Feed System on Meat Fatty Acid Profile

### 6.1. Saturated Fatty Acids

Despite potential protective effects, ruminant meat is a major contributor to SFA intake. The average UK adult intake of SFAs is 13.1% (aged 65–74 years) and 14.6% (aged 75 years and older) of total energy intake, which is higher than the recommended daily allowance of <11% [110,111]. Whilst SFA consumption needs to be reduced, addressing this through meat consumption habits is complicated. Organic meat is inconsistently different from conventional meat with regards to SFAs, potentially based on the within-system variation in the amount of forage in diets or the degree of animal fatness, as organic or grass-fed beef is frequently leaner than conventional beef or beef from intensive systems [39,112]. Ribas-Agustí et al. [40] found organic retail beef has 8% fewer SFAs compared with conventional beef and Bjorklund et al. [113] reported organic beef had 23% fewer SFAs than conventional beef from steers of the same breed. One study differentiating pasture-based and non-pasture systems for Angus heifers, found grass-finished cooked beef had 30% fewer SFAs compared to concentrate finished beef [114]. Additionally, the review by Daley et al. [115] reports no difference in total SFAs between grain-fed and pasture-based beef, but higher concentrations of myristic (C14:0) and palmitic acids (C16:0) (thought to have a greater impact on serum cholesterol [115]) in intensive grain-fed beef and lower concentrations of stearic acid (thought to have a neutral impact on serum cholesterol [115]) than in grass-fed beef, suggesting that grass-fed may have a nutritionally favourable SFA profile than grain-fed beef. Complicating the lack of clear difference in meat SFA content based on feed system, a meta-analysis found no difference in the proportion of SFAs between the organic and conventional systems [39]. In following dietary recommendations to reduce SFA intake overall, there is some evidence that switching to organic may reduce total SFA intake (although inconsistent between studies), but it seems that consumption of meat from pasture-based ruminant systems contributes to the intake of a more favourable SFA profile (although overall SFA might be similar). 

### 6.2. Monounsaturated Fatty Acids

Conventional beef has been shown to have more (by both concentration and proportion) MUFA than organic and/or grazing-based alternatives [35,39,116,117,118]. It is however unclear why this difference occurs (potentially to do with the supply of oleic acids from conventional diets and/or the de novo synthesis of OA in the muscle), and there are no known reports associating the higher MUFA in conventional beef on human nutrition and health; further research is required.

Despite organic and/or grass-fed meat having less overall MUFA, it sometimes contains more VA (t11 C18:1) [115,117], resulting in greater de novo synthesis of the beneficial CLA9 within body tissues. As discussed, CLA9 has been associated with positive health outcomes, suggesting that any increase in this FA, could positively impact human health, although, again, more research is needed to assess this.

### 6.3. Polyunsaturated Fatty Acids

Consistently across studies, total PUFA content is higher in grass-fed meat, followed by organic meat [39,114,117,118,119]. A meta-analysis by Średnicka-Tober et al. [39] found organic meat to have around 23% more total PUFAs than conventional counterparts. However, this elevated concentration of PUFA seems to be at the expense of MUFA, rather than SFA. So, whilst an increase in PUFA, in theory, may have positive outcomes on human health, this could be negated as it does not directly align with the nutritional recommendation to replace SFA with cis-MUFA and cis-PUFA [67]. 

#### 6.3.1. Omega-3 Fatty Acids

Beef is a source of essential long-chain omega-3 fatty acids, which are often under-consumed in the human diet and meat products are one of the main sources of these FAs (while oily fish probably represents their most widely recommended source) [117]. Consumption of very-long-chain (VLC) *n*-3 PUFA reduces the risk of cardiovascular disease, as well as demonstrating reduced arrhythmia, blood pressure, inflammation, platelet sensitivity and risk of dementia, contributing to foetal brain development and delaying mental cognition decline in elderly men [120,121].

Analysis of beef from Aberdeen Angus cattle fed: (a) only grass, (b) grass silage and concentrates or c) only concentrates found grass-only feeding produced meat significantly higher in *n*-3 PUFA, including ALA and DPA [114] and EPA and DHA [105]. Consuming ruminant meat could be a good method of increasing population *n*-3 and VLC *n*-3 intakes [122,123] since organic and pasture-fed beef has more *n*-3 and VLC *n*-3, contributing to higher *n*-3 intakes for the consumer [35,39,116,117,124]. Additionally, certified 100% pasture-fed beef could qualify as ‘sources of long-chain *n*-3′ (pasture-fed: 41 mg VLC/100 g steak, conventional: 28 mg VLC/100 g steak) [117], with more than 40 mg VLC *n*-3 per 100 g food, as regulated by the European Food Standards [125]. Whilst no cohort study examining the impact of consuming organic and/or pasture-fed meat on human health has been conducted, evidence points towards an increase in *n*-3 intakes from pasture-based beef, which could be highly relevant for consumers who do not consume oily fish (thus meat being their major source of *n*-3).

#### 6.3.2. Omega-6 Fatty Acids

There seems to be a mix of results for omega-6 content when comparing organic and grass-fed beef to conventional, with some studies finding more LA in organic [35,40] and others in conventional beef [116,117]; however, in nearly all publications, the difference in LA between the systems is marginal, suggesting that management (and thus, potentially diet) has very little impact on the LA or total *n*-6 concentration in beef. 

#### 6.3.3. Omega-6:Omega-3 Ratio

Many studies show beef from grazing systems have lower *n*-6:*n*-3 ratios compared with maize silage [105] and grain-based feeding [39,42,113,117,118,126]. The evidence strongly suggests that the more fresh forage in ruminant feeding, the lower the *n*-6:*n*-3 ratio in their diet, contributing to a reduced ratio in the resulting beef. In a comparison of the fatty acid content of sirloin steaks from UK retail outlets, the *n*-6:*n*-3 ratio was lower in organic (1.49) compared to conventional (2.78) meat [116]. A study comparing meat from crossbred steers reared on a pasture-based diet compared with a corn/maize grain diet found that pasture-fed beef produced a lower LA:ALA ratio (3.9 vs. 6.7, respectively) [119], which is reflective of the *n*-6:*n*-3 ratio. Similarly, Lenighan et al. [114] investigated meat quality from heifers finished under three different diets, analysing the fat quality in cooked meat and found the same pattern with an *n*-6:*n*-3 ratio from grass-fed finished beef at 1.2 and concentrate finished at 4.2, while the grass silage and concentrate finished beef split the difference at 1.9. A US study considered meat quality from feed-lot (20% forage), organic (>30% forage) and grass-fed (100% forage) finished steers and found a much greater difference in *n*-6:*n*-3 ratio (20.7, 14.4 and 1.5, respectively) [113]. Berthelot and Gruffat [105] identified a similar pattern in an analysis of 46 publications, finding that *n*-6:*n*-3 ratios decreased with diet-type changes toward more grass intake (concentrate = 10.0; corn-silage = 8.7; concentrate-forage = 5.8; grass-based = 2.4). This is potentially reflective of the US maize-based ruminant diets, both as silage and dry corn, which have much higher LA concentrations than grass-based alternatives [127,128]. This pattern indicates that when cattle are typically fed diets with more *n*-6 (i.e., intensive growing/finishing feeding systems), there will be a much greater decrease in the *n*-6:*n*-3 ratio when switching (or comparing) to pasture-fed beef, thus having more of an impact on the overall ratio consumed and potentially nutritional and health attributes of the meat. Interestingly, there do not appear to be major differences in meat FA profile from cattle grazing different sward types [129], further demonstrating that the majority of the benefit to meat FA profile comes from the partial or full replacement of grain with fresh, preferably grazed, pasture or forages.

#### 6.3.4. CLA

There are many studies showing CLA9 concentrations in beef increase as forage content increases in the ruminant diet (similarly to omega-3) [117,130]; however, increases in red meat consumption (without distinguishing between ruminant diets) have been linked to an increased risk of premature death and colorectal cancer [9]. The health impacts from increasing consumption of ruminant produce with high CLA9 are difficult to distinguish given this overall assessment of red meat, similar to most fatty acids discussed in this review.

## 7. Human Health Implications

Increasing the amount of forage in the ruminant diet results in a higher proportion of *n*-3 and lower *n*-6:*n*-3 ratio, which is both highly relevant for human health; however, with this meat forming a very low proportion of the typical UK diet, would switching to forage-fed beef significantly impact population health? To answer this would require: (i) evaluating/modelling the impacts of switching to forage-fed beef on intakes of nutritionally relevant FAs and groups in a typical diet, and (ii) conducting long-term human intervention dietary trials. The present study attempts the former considering the contribution to recommended dietary intakes (RDI), using beef fatty acid concentrations from studies evaluating composition from different feeding systems applied to typical red meat consumption or following two recommended consumption guidelines. 

As discussed, there are many factors that drive changes in fat composition. These differences in the composition will ultimately impact nutrient supply to consumers of conventional, organic and grass-fed meat. For this review, we modelled examples using three consumption scenarios: (i) average UK population National Diet and Nutrition Survey (NDNS) (<128 g/week) [131] red meat consumption, (ii) red meat consumption suggested by the UK National Health Service (NHS) (<490 g/week) [132] and (iii) red meat consumption suggested by the Eat Lancet Report (<98 g/week) [7]. The average fat content of beef was assumed to be 10.61 g/100 g, as per McCance and Widdowson’s composition of foods integrated dataset [38], from 23 categories of cooked beef cuts. To transform FA contents from raw values reported in the literature to cooked values, 23 raw beef cuts and the equivalent cooked cuts were compared in the same database and the correction factor of fat content for cooked:raw was estimated to be 10.61:9.52 = 1.115. The conversion factor to determine total fatty acids in fat was estimated as 0.935 according to McCance and Widowson’s 7th summary edition [38]. In the first scenario, intakes of beef fat (g/day) were recorded fat intakes from the “beef, veal and dishes” food category in NDNS. In the second and third scenario, fat intakes were calculated by converting recommended beef intakes to fat intakes using an average content of 10.61 g fat/100 g cooked beef (from 23 cooked beef foods in the McCance and Widdowson’s composition of foods integrated dataset [38]); and were 52.0 g fat/week and 10.4 g fat/week, respectively. Table 1 describes the sources of meat fatty acid profiles used in these scenarios, which included three retail studies and four independent animal experiments investigating nutritional differences between feed and management systems, using IM adipose tissue and similar data units. The limited number of papers presented in this table demonstrates the lack of comparable studies in the literature. 

Table 2 shows predicted nutrient intakes averaged across age and sex, with the three different scenarios, under four different feeding systems: (i) 100% pasture-based (PB), (ii) organic (ORG), (iii) conventional (CON) and (iv) intensive grain-fed (INT). The full nutrient intakes from the different scenarios, expressed as % RDIs categorised by age and sex, are included in Supplementary Materials (Tables S1–S6).

The nutrient intakes (Table 2) resulting from this modelling exercise are presented as the proportion of daily recommended intakes (RDI) supplied from each of the consumption scenarios (NDNS, NHS and Eat Lancet). RDIs used in the present study were in line with the Scientific Advisory Committee on Nutrition (SACN) [67]. These are: SFA, <10% energy intake (EI); trans-fat, <2% EI; MUFA, 12% EI; cis-PUFA, 6% EI; long-chain *n*-3 PUFA, 200–450 mg/day; *n*-6, <10% EI; ALNA, >0.2% EI; LA, >1% EI. Energy intakes for the different demographics were in line with SACN reference values [133]. For many of the fatty acids listed, red meat is not the primary source of these nutrients, making some of the differences minimal; however, the most notable differences are for total *n*-3, ALA and EPA + DHA, which diet and nutrition literature suggest are fatty acids that should increase in the human diet. Based on NHS guidelines, and assuming all ‘red meat’ was beef (not pork or lamb and not processed products), 82.6% RDI of *n*-3 could come from pasture-based beef, compared to 27.1% from intensive grain-fed, 22.3% from conventional, and 32.0% from organic beef (Table 2). Though the overall proportion of RDI is lower, the same pattern exists for ALA (38.9% from PB, 9.9% INT, 10.2% CON and 15.3% ORG) and EPA + DPA (66.4% from PB, 29.4% INT, 18.4% CON and 14.9% ORG). Although there are differences between the proportion of these fatty acid RDIs coming from organic, conventional and intensive beef, it seems that consumption of pasture-based beef would provide a substantially higher contribution of some beneficial FAs in human diets. Given that the difference between the undesirable fatty acids is minimal (SFAs and trans FAs) from a nutrition perspective switching to pasture-based beef seems to be a reliable method of increasing desirable *n*-3 FAs in human diets. 

The study also considered guidelines in the Eat Lancet report [7] to reflect dietary recommendations derived from concerns over the ecological consequences of animal production systems. These recommendations skew to the extreme of plant-based diets while still allowing for a small amount of meat consumption. Even at this much lower consumption of red meat at 14 g/day, 16.5% RDI of *n*-3 could be achieved from pasture-based beef, compared to 5.4% of intensively reared, 4.5% conventional and 6.4% of organic beef). This is similarly true for ALA (7.8% from PB, 2.0% from INT and CON and 3.1% from ORG) and EPA + DHA (13.3% from PB, 5.9% from INT, 3.7% from CON and 3.0% from ORG). While discussing the nutritional and dietary viability of extreme reductions in meat consumption are beyond the scope of this paper, the evidence demonstrates that even for the predominately plant-based diet recommended by the Eat Lancet report, where less of the RDI of fatty acids will be derived from meat, the feeding system used to produce this meat can still play a role on daily intakes of essential FAs, with pasture-based meat increasing these intakes. 

Given that findings in Table 2 and Tables S1–S6 make several assumptions and are based on the average fat contents across different cuts, they provide a generic picture of potential nutritional implications, although contributions may substantially change with the consumption of meat with different fat content. These scenarios are not included in the present paper but can be derived using the formula: contribution to % RDI of *x* FA when cooked beef cut with *z*% fat content is consumed = (*z*/10.61) × % RDI contribution towards *x* FA presented in the tables in the present study. For example, the contribution of the different FAs for lean, roasted medium-rare topside cuts (with *z* = 5.1% fat content [38]), values in the tables should be multiplied with a correction factor of *z*/10.61 = 5.1/10.61 = 0.4807. On the contrary, for a fatty pot-roasted flank (with *z* = 22.3% fat content [38]), all values in the tables should be multiplied with a correction factor of *z*/10.61 = 22.3/10.61 = 2.1018

In the present study, estimated values also assume a correction factor for the fat content of cooked vs. raw beef cuts, to account for the increase in fat content due to moisture loss during cooking [38]; however, this does not account for any potential effect of the cooking method, known to influence the FA profile consumed, primarily via the addition of fats during cooking (e.g., pan-fried rib-eye steaks) or temperature and duration of cooking [67]. The RDIs used in the present study, as well as the recommended energy intakes, were in line with the Scientific Advisory Committee on Nutrition (SACN), as mentioned earlier [67]. It is recommended that future comparative work should account for the differences between countries and organisations on nutrient reference values and recommended intakes, as these would affect the relative contribution from beef. 

## 8. Conclusions

This review set out to examine if organic and/or pasture-based beef confers nutritional benefits compared to conventionally produced beef. The evidence suggests the fatty acid profile of beef meat produced under pasture-based diets has a more nutritionally desirable fatty acid profile than intensively and conventionally reared beef, and to a lesser extent, maybe even compared with organically reared beef. Although some beneficial fatty acids are more prevalent in some analyses of organic beef compared with conventional, the driving force behind the improved fatty acid profile is the forage proportion in the diet. This is particularly evident in the much higher percentage of total omega-3 and long-chain fatty acids EPA + DHA from pasture-based diets compared to organically reared beef. The literature review and analysis of predicted fatty acid intakes based on recorded/recommended meat intakes and the RDI for the different FAs and FA groups demonstrate that the average consumer would receive more of the beneficial fatty acids for human health through consumption of pasture-based beef, produced either organically or conventionally.

The impact that human consumption habits have on health will continue to gain interest over time. Yet, it is important to consider that the individual foods and nutrients do not directly affect human health in isolation, but feature as part of the whole diet, which interact with many other factors (exercise, socio-economic status, access to healthcare, etc.) that ultimately determine health. Therefore, switching to pasture-fed and/or organic beef will provide more of the beneficial nutrients, but as to whether that will directly impact human health requires analysis in human studies and consideration of several factors (nutritional and other) beyond beef fat quality.

## Figures and Tables

**Table 1:** Feed system, meat source and country of the seven studies included in the data analysis of fatty acid consumption from beef.

Source	Feed System ^1^				Meat Sources	Country
Alfaia, Alves, Martins, Costa, Fontes, Lemos, Bessa and Prates [118]	PB			INT	Alentejano purebred bulls	Portugal
Berthelot and Gruffat [105]	PB		CON	INT	Beef cattle	France
Bjorklund, Heins, DiCostanzo and Chester-Jones [113]	PB	ORG	CON		Crossbred Dairy Bulls	USA
Butler, Ali, Oladokun, Wang and Davis [117]	PB	ORG	CON		Supermarket & Farms (grass-fed)	UK
Descalzo, Insani, Biolatto, Sancho, García, Pensel and Josifovich [119]	PB			INT	Crossbred Steers	Argentina
Kamihiro, Stergiadis, Leifert, Eyre and Butler [116]		ORG	CON		Supermarket	UK
Łozicki, Dymnicka, Arkuszewska and Pustkowiak [35]		ORG	CON		Hereford bulls	Poland
Ribas-Agustí, Díaz, Sárraga, García-Regueiro and Castellari [40]		ORG	CON		Supermarket	Spain

^1^ PB: pasture-based; ORG: organic; CON: conventional; INT: intensive grain-fed.

**Table 2:** Predicted fatty acid intakes from beef from four different feeding systems (Intensive: INT; Conventional: CON; Organic: ORG; Pasture-Based: PB) as a percentage of recommended daily intake (RDI) ^1^, based on three different intake scenarios (NDNS, NHS, EAT LANCET) averaged across age and sex; assuming beef fat content of 10.61 g/100 g (averaged from 23 categories of cooked beef cuts) and beef fat to be 93.5% fatty acids, according to the McCance and Widdowson’s composition of foods integrated dataset [38].

Recorded Beef Fat Intakes NDNS ^2^ (21 g/Week, 3 g/Day)				
% RDI	Intensive	Conventional	Organic	Pasture-Based
SFA	3.6	3.6	3.5	3.3
Trans	1.5	1.8	1.2	1.1
MUFA	2.7	3.2	3.0	2.5
cis-PUFA		0.4	0.3	0.2
*n*-3	6.7	5.5	8.0	20.6
*n*-6	1.1	0.4	0.4	0.7
ALA	2.3	2.4	3.6	9.2
LA	6.7	3.8	3.8	4.5
EPA + DHA	7.3	4.6	3.7	16.5
**Intakes NHS ^3^ (490 g beef/week, 70 g/day)**				
	**Intensive**	**Conventional**	**Organic**	**Pasture-based**
SFA	14.4	14.2	14.0	13.0
Trans	6.2	7.6	5.0	4.8
MUFA	11.3	13.5	12.8	10.5
cis-PUFA		1.6	1.4	0.7
*n*-3	27.1	22.3	32.0	82.6
*n*-6	4.5	1.9	1.7	2.9
ALA	9.9	10.2	15.3	38.9
LA	28.4	16.3	16.2	19.2
EPA + DHA	29.4	18.4	14.9	66.4
**Intakes EAT LANCET ^4^ (98 g beef/week, 14 g/day)**				
	**Intensive**	**Conventional**	**Organic**	**Pasture-Based**
SFA	2.9	2.8	2.8	2.6
Trans	1.2	1.5	1.0	1.0
MUFA	2.3	2.7	2.6	2.1
cis-PUFA		0.3	0.3	0.1
*n*-3	5.4	4.5	6.4	16.5
*n*-6	0.9	0.4	0.3	0.6
ALA	2.0	2.0	3.1	7.8
LA	5.7	3.3	3.2	3.8
EPA + DHA	5.9	3.7	3.0	13.3

^1^ RDIs in line with the Scientific Advisory Committee on Nutrition (SACN) [67]: SFA <10% energy intake (EI); trans-fat <2% EI; MUFA 12% EI; cis-PUFA = 6% EI; long-chain *n*-3 PUFA = 200–450 mg/day; *n*-6 <10% EI; ALNA >0.2% EI; LA,>1% EI. ^2^ NDNS: National Diet and Nutrition Survey Years 9-11 [131]; ^3^ NHS: UK National Health Service [132]; ^4^ EAT LANCET: Eat Lancet report [7].

## Supplementary Materials

The following are available online at https://www.mdpi.com/article/10.3390/foods11050646/s1, Table S1: Predicted fatty acid intakes from beef produced in four different beef feed systems (Intensive, INT; Conventional, CON; Organic, ORG; Pasture-Based, PB) as a percentage of recommended daily intake (RDI) across sex and age based on beef fat intakes from the National Diet and Nutrition Survey; assuming average fat content of beef been 10.61 g/100 g (averaged from 23 categories of cooked beef cuts), and fatty acid content of beef fat to be 93.5%, according to the McCance and Widdowson’s composition of foods integrated dataset; Table S2: Predicted fatty acid intakes from beef produced in four different beef feed systems (Intensive, INT; Conventional, CON; Organic, ORG; Pasture-Based, PB) as a percentage of recommended daily intake (RDI) across sex and age based on beef fat intakes from the National Diet and Nutrition Survey; assuming average fat content of beef been 10.61 g/100 g (averaged from 23 categories of cooked beef cuts), and fatty acid content of beef fat to be 93.5%, according to the McCance and Widdowson’s composition of foods integrated dataset; Table S3: Predicted fatty acid intakes from beef produced in four different beef feed systems (Intensive, INT; Conventional, CON; Organic, ORG; Pasture-Based, PB) as a percentage of recommended daily intake (RDI) across sex and age based on the NHS: UK National Health Service recommendations of 490 g beef/week (70 g/day); assuming average fat content of beef been 10.61 g/100 g (averaged from 23 categories of cooked beef cuts), and fatty acid content of beef fat to be 93.5%, according to the McCance and Widdowson’s composition of foods integrated dataset; Table S4: Predicted fatty acid intakes from beef produced in four different beef feed systems (Intensive, INT; Conventional, CON; Organic, ORG; Pasture-Based, PB) as a percentage of recommended daily intake (RDI) across sex and age based on the NHS: UK National Health Service recommendations of 490 g beef/week (70 g/day); assuming average fat content of beef been 10.61 g/100 g (averaged from 23 categories of cooked beef cuts), and fatty acid content of beef fat to be 93.5%, according to the McCance and Widdowson’s composition of foods integrated dataset; Table S5: Predicted fatty acid intakes from beef produced in four different beef feed systems (Intensive, INT; Conventional, CON; Organic, ORG; Pasture-Based, PB) as a percentage of recommended daily intake (RDI) across sex and age based on the EAT LANCET: Eat Lancet report of 98 g beef/week (14 g/day); assuming average fat content of beef been 10.61 g/100 g (averaged from 23 categories of cooked beef cuts), and fatty acid content of beef fat to be 93.5%, according to the McCance and Widdowson’s composition of foods integrated dataset; Table S6: Predicted fatty acid intakes from beef produced in four different beef feed systems (Intensive, INT; Conventional, CON; Organic, ORG; Pasture-Based, PB) as a percentage of recommended daily intake (RDI) across sex and age based on the EAT LANCET: Eat Lancet report of 98 g beef/week (14 g/day); assuming average fat content of beef been 10.61 g/100 g (averaged from 23 categories of cooked beef cuts), and fatty acid content of beef fat to be 93.5%, according to the McCance and Widdowson’s composition of foods integrated dataset.
