# Supplementary material for: Nutritional Benefits from Fatty Acids in Organic and Grass-Fed Beef
Source: Foods. 2022 Feb 23;11(5):646. doi: 10.3390/foods11050646 (PMC8909876; doi:10.3390/foods11050646)
Supplement: Supplementary file 1 [file foods-11-00646-s001.zip › foods-1563516-supplementary.pdf]

Supplementary Materials

Table S1. Predicted fatty acid intakes from beef produced in four different beef feed systems (Intensive, INT; Conventional, CON; Organic, ORG; Pasture-Based, PB) as a percentage of recommended daily intake (RDI)[1] across sex and age based on beef fat intakes from the National Diet and Nutrition Survey [1]; assuming average fat content of beef been 10.61 g/100g (averaged from 23 categories of cooked beef cuts), and fatty acid content of beef fat to be 93.5%, according to the McCance and Widdowson's composition of foods integrated dataset [2].

	SFA[2]				Trans FA[3]				MUFA[4]				cis-PUFA[5]			
Demographic group	INT	CON	ORG	PB	INT	CON	ORG	PB	INT	CON	ORG	PB	INT	CON	ORG	PB
Children 1.5-3					1.4	1.7	1.1	1.0	2.5	3.0	2.8	2.3		0.3	0.3	0.2
Boys 4-10	3.2	3.2	3.1	2.9	1.3	1.6	1.0	1.0	2.4	2.8	2.7	2.2		0.3	0.3	0.1
Girls 4-10	3.0	3.0	3.0	2.7	1.2	1.5	1.0	1.0	2.3	2.7	2.6	2.1		0.3	0.3	0.1
Children 4-10	3.1	3.1	3.0	2.8	1.3	1.6	1.0	1.0	2.3	2.8	2.6	2.2		0.3	0.3	0.1
Boys 11-18	2.7	2.7	2.7	2.5	1.1	1.4	0.9	0.9	2.0	2.4	2.3	1.9		0.3	0.2	0.1
Girls 11-18	2.8	2.8	2.7	2.5	1.2	1.4	0.9	0.9	2.1	2.5	2.4	1.9		0.3	0.3	0.1
Children 11-18	2.8	2.7	2.7	2.5	1.1	1.4	0.9	0.9	2.1	2.5	2.3	1.9		0.3	0.2	0.1
Men 19-64	3.7	3.7	3.6	3.4	1.5	1.9	1.2	1.2	2.8	3.3	3.1	2.6		0.4	0.3	0.2
Women 19-64	3.7	3.7	3.6	3.4	1.5	1.9	1.2	1.2	2.8	3.3	3.1	2.6		0.4	0.3	0.2
Adults 19-64	3.7	3.7	3.6	3.4	1.5	1.9	1.2	1.2	2.8	3.3	3.1	2.6		0.4	0.3	0.2
Men 65+	4.0	3.9	3.9	3.6	1.6	2.0	1.3	1.2	3.0	3.5	3.3	2.8		0.4	0.4	0.2
Women 65+	4.1	4.1	4.0	3.7	1.7	2.1	1.3	1.3	3.1	3.6	3.5	2.9		0.4	0.4	0.2
Adults 65+	4.0	4.0	3.9	3.6	1.6	2.0	1.3	1.3	3.0	3.6	3.4	2.8		0.4	0.4	0.2
Men 65-74	4.0	4.0	3.9	3.6	1.6	2.0	1.3	1.3	3.0	3.6	3.4	2.8		0.4	0.4	0.2
Women 65-74	4.1	4.0	4.0	3.7	1.7	2.0	1.3	1.3	3.0	3.6	3.4	2.8		0.4	0.4	0.2
Adults 65-74	4.0	4.0	3.9	3.6	1.7	2.0	1.3	1.3	3.0	3.6	3.4	2.8		0.4	0.4	0.2
Men 75+	3.8	3.8	3.7	3.5	1.6	1.9	1.2	1.2	2.8	3.4	3.2	2.6		0.4	0.3	0.2
Women 75+	4.1	4.0	4.0	3.7	1.7	2.0	1.3	1.3	3.0	3.6	3.4	2.8		0.4	0.4	0.2
Adults 75+	3.9	3.8	3.8	3.5	1.6	1.9	1.3	1.2	2.9	3.4	3.3	2.7		0.4	0.3	0.2

[1] RDIs from the Scientific Advisory Committee on Nutrition (SACN) [3]: SFA, <10% energy intake (EI); trans-fat, <2% EI; MUFA, 12 % EI; cis-PUFA, 6% EI; and EI for the different demographics as in SACN reference values [4]. [2] SFA, Saturated Fatty Acids. [3] Trans FA, Trans- Fatty Acids. [4] MUFA; Monounsaturated Fatty Acids. [5] cis-PUFA, cis-Polyunsaturated Fatty Acids.

Table S2. Predicted fatty acid intakes from beef produced in four different beef feed systems (Intensive, INT; Conventional, CON; Organic, ORG; Pasture-Based, PB) as a percentage of recommended daily intake (RDI)[1] across sex and age based on beef fat intakes from the National Diet and Nutrition Survey [1]; assuming average fat content of beef been 10.61 g/100g (averaged from 23 categories of cooked beef cuts), and fatty acid content of beef fat to be 93.5%, according to the McCance and Widdowson's composition of foods integrated dataset [2].

	Omega-3				Omega-6				ALA[1]				LA[2]				EPA+DHA[3]			
Demographic group	INT	CON	ORG	PB	INT	CON	ORG	PB	INT	CON	ORG	PB	INT	CON	ORG	PB	INT	CON	ORG	PB
Children 1.5-3	3.0	2.5	3.5	9.1	1.0	0.4	0.4	0.6	2.2	2.2	3.4	8.5	6.2	3.6	3.5	4.2	3.2	2.0	1.6	7.3
Boys 4-10	4.9	4.0	5.7	14.8	1.0	0.4	0.4	0.6	2.1	2.2	3.2	8.2	6.0	3.4	3.4	4.0	5.3	3.3	2.7	11.9
Girls 4-10	4.3	3.5	5.1	13.1	0.9	0.4	0.3	0.6	2.0	2.0	3.1	7.8	5.7	3.3	3.2	3.8	4.7	2.9	2.4	10.6
Children 4-10	4.6	3.8	5.4	14.0	0.9	0.4	0.3	0.6	2.0	2.1	3.1	8.0	5.8	3.4	3.3	4.0	5.0	3.1	2.5	11.2
Boys 11-18	6.6	5.5	7.8	20.2	0.8	0.3	0.3	0.5	1.8	1.8	2.7	7.0	5.1	2.9	2.9	3.5	7.2	4.5	3.6	16.3
Girls 11-18	5.9	4.8	6.9	17.9	0.8	0.3	0.3	0.5	1.8	1.9	2.8	7.2	5.3	3.0	3.0	3.5	6.4	4.0	3.2	14.4
Children 11-18	6.3	5.2	7.4	19.1	0.8	0.3	0.3	0.5	1.8	1.9	2.8	7.1	5.2	3.0	2.9	3.5	6.8	4.2	3.4	15.3
Men 19-64	9.0	7.4	10.6	27.4	1.1	0.5	0.4	0.7	2.4	2.5	3.7	9.5	7.0	4.0	4.0	4.7	9.8	6.1	4.9	22.0
Women 19-64	7.2	5.9	8.5	22.0	1.1	0.5	0.4	0.7	2.4	2.5	3.8	9.6	7.0	4.0	4.0	4.7	7.8	4.9	4.0	17.7
Adults 19-64	8.1	6.7	9.6	24.7	1.1	0.5	0.4	0.7	2.4	2.5	3.7	9.5	7.0	4.0	4.0	4.7	8.8	5.5	4.4	19.8
Men 65+	8.4	6.9	9.9	25.5	1.2	0.5	0.4	0.8	2.6	2.7	4.0	10.2	7.5	4.3	4.2	5.0	9.1	5.7	4.6	20.5
Women 65+	7.0	5.8	8.3	21.3	1.2	0.5	0.5	0.8	2.7	2.8	4.1	10.5	7.7	4.4	4.4	5.2	7.6	4.7	3.8	17.2
Adults 65+	7.6	6.3	9.0	23.2	1.2	0.5	0.4	0.8	2.6	2.7	4.0	10.3	7.5	4.3	4.3	5.1	8.3	5.2	4.2	18.7
Men 65-74	8.5	7.0	10.1	26.0	1.2	0.5	0.4	0.8	2.6	2.7	4.0	10.3	7.5	4.3	4.3	5.1	9.3	5.8	4.7	20.9
Women 65-74	7.1	5.8	8.3	21.5	1.2	0.5	0.5	0.8	2.7	2.7	4.1	10.4	7.6	4.4	4.3	5.1	7.7	4.8	3.9	17.3
Adults 65-74	7.8	6.4	9.2	23.6	1.2	0.5	0.4	0.8	2.6	2.7	4.0	10.3	7.5	4.3	4.3	5.1	8.4	5.3	4.3	19.0
Men 75+	7.9	6.5	9.4	24.2	1.1	0.5	0.4	0.7	2.5	2.6	3.8	9.8	7.1	4.1	4.1	4.8	8.6	5.4	4.4	19.5
Women 75+	6.8	5.6	8.0	20.7	1.2	0.5	0.5	0.8	2.7	2.7	4.1	10.4	7.6	4.4	4.3	5.1	7.4	4.6	3.7	16.6
Adults 75+	7.3	6.0	8.6	22.2	1.2	0.5	0.4	0.7	2.5	2.6	3.9	9.9	7.3	4.2	4.1	4.9	7.9	4.9	4.0	17.8

[1] RDIs from the Scientific Advisory Committee on Nutrition (SACN) [3]: long-chain n-3 PUFA, 200-450 mg/day; n-6, <10% EI; ALNA, >0.2% EI; LA, >1% EI; and EI for the different demographics as in SACN reference values [4]. [1] ALA; alpha-linolenic acid. [2] LA; alpha-linolenic acid. [3] EPA, eicosapentaenoic acid and DHA, docosahexaenoic acid.

Table S3. Predicted fatty acid intakes from beef produced in four different beef feed systems (Intensive, INT; Conventional, CON; Organic, ORG; Pasture-Based, PB) as a percentage of recommended daily intake (RDI)[1] across sex and age based on the NHS: UK National Health Service recommendations [5] of 490 g beef/week (70 g/day); assuming average fat content of beef been 10.61 g/100g (averaged from 23 categories of cooked beef cuts), and fatty acid content of beef fat to be 93.5%, according to the McCance and Widdowson's composition of foods integrated dataset [2].

	SFA[2]				Trans FA[3]				MUFA[4]				cis-PUFA[5]			
Demographic group	INT	CON	ORG	PB	INT	CON	ORG	PB	INT	CON	ORG	PB	INT	CON	ORG	PB
Children 1.5-3					12.4	15.1	9.9	9.5	22.5	26.8	25.4	21.0		3.2	2.7	1.4
Boys 4-10	17.9	17.7	17.4	16.2	7.3	9.0	5.8	5.6	13.3	15.8	15.0	12.4		1.9	1.6	0.8
Girls 4-10	19.2	18.9	18.6	17.3	7.8	9.6	6.2	6.0	14.2	16.9	16.1	13.3		2.0	1.7	0.9
Children 4-10	18.5	18.3	18.0	16.7	7.6	9.3	6.0	5.8	13.7	16.4	15.5	12.8		1.9	1.7	0.9
Boys 11-18	11.2	11.0	10.8	10.1	4.6	5.6	3.6	3.5	8.3	9.9	9.4	7.7		1.2	1.0	0.5
Girls 11-18	13.0	12.9	12.6	11.7	5.3	6.5	4.2	4.1	9.6	11.5	10.9	9.0		1.4	1.2	0.6
Children 11-18	12.0	11.9	11.7	10.9	4.9	6.0	3.9	3.8	8.9	10.6	10.1	8.3		1.3	1.1	0.6
Men 19-64	11.2	11.1	10.9	10.2	4.6	5.6	3.7	3.5	8.3	9.9	9.4	7.8		1.2	1.0	0.5
Women 19-64	14.1	13.9	13.7	12.7	5.8	7.0	4.6	4.4	10.4	12.4	11.8	9.7		1.5	1.3	0.7
Adults 19-64	12.5	12.4	12.1	11.3	5.1	6.3	4.1	3.9	9.3	11.1	10.5	8.7		1.3	1.1	0.6
Men 65+	12.9	12.8	12.5	11.7	5.3	6.5	4.2	4.0	9.6	11.4	10.8	8.9		1.4	1.2	0.6
Women 65+	16.0	15.8	15.5	14.4	6.5	8.0	5.2	5.0	11.8	14.1	13.4	11.1		1.7	1.4	0.7
Adults 65+	14.3	14.1	13.9	12.9	5.8	7.1	4.7	4.5	10.6	12.6	12.0	9.9		1.5	1.3	0.7
Men 65-74	12.8	12.6	12.4	11.5	5.2	6.4	4.2	4.0	9.5	11.3	10.7	8.9		1.3	1.1	0.6
Women 65-74	15.7	15.5	15.2	14.1	6.4	7.8	5.1	4.9	11.6	13.8	13.1	10.8		1.6	1.4	0.7
Adults 65-74	14.1	13.9	13.7	12.7	5.8	7.0	4.6	4.4	10.4	12.4	11.8	9.7		1.5	1.3	0.7
Men 75+	13.1	12.9	12.7	11.8	5.3	6.5	4.3	4.1	9.7	11.5	10.9	9.0		1.4	1.2	0.6
Women 75+	16.3	16.1	15.8	14.7	6.7	8.1	5.3	5.1	12.1	14.4	13.6	11.3		1.7	1.5	0.8
Adults 75+	14.5	14.3	14.1	13.1	5.9	7.2	4.7	4.5	10.8	12.8	12.1	10.0		1.5	1.3	0.7

[1] RDIs from the Scientific Advisory Committee on Nutrition (SACN) [3]: SFA, <10% energy intake (EI); trans-fat, <2% EI; MUFA, 12 % EI; cis-PUFA, 6% EI; and EI for the different demographics as in SACN reference values [4]. [2] SFA, Saturated Fatty Acids. [3] Trans FA, Trans- Fatty Acids. [4] MUFA; Monounsaturated Fatty Acids. [5] cis-PUFA, cis-Polyunsaturated Fatty Acids.

Table S4. Predicted fatty acid intakes from beef produced in four different beef feed systems (Intensive, INT; Conventional, CON; Organic, ORG; Pasture-Based, PB) as a percentage of recommended daily intake (RDI)[1] across sex and age based on the NHS: UK National Health Service recommendations [5] of 490 g beef/week (70 g/day); assuming average fat content of beef been 10.61 g/100g (averaged from 23 categories of cooked beef cuts), and fatty acid content of beef fat to be 93.5%, according to the McCance and Widdowson's composition of foods integrated dataset [2].

	Omega-3				Omega-6				ALA[1]				LA[2]				EPA+DHA[3]			
Demographic group	INT	CON	ORG	PB	INT	CON	ORG	PB	INT	CON	ORG	PB	INT	CON	ORG	PB	INT	CON	ORG	PB
Children 1.5-3	27.1	22.3	32.0	82.6	9.0	3.7	3.4	5.8	19.7	20.3	30.4	77.3	56.5	32.4	32.1	38.2	29.4	18.4	14.9	66.4
Boys 4-10	27.1	22.3	32.0	82.6	5.3	2.2	2.0	3.4	11.7	12.0	18.0	45.7	33.4	19.1	19.0	22.6	29.4	18.4	14.9	66.4
Girls 4-10	27.1	22.3	32.0	82.6	5.7	2.3	2.1	3.7	12.5	12.9	19.2	48.9	35.7	20.5	20.3	24.1	29.4	18.4	14.9	66.4
Children 4-10	27.1	22.3	32.0	82.6	5.5	2.3	2.1	3.5	12.1	12.4	18.6	47.2	34.5	19.8	19.6	23.3	29.4	18.4	14.9	66.4
Boys 11-18	27.1	22.3	32.0	82.6	3.3	1.4	1.2	2.1	7.3	7.5	11.2	28.5	20.9	11.9	11.9	14.1	29.4	18.4	14.9	66.4
Girls 11-18	27.1	22.3	32.0	82.6	3.9	1.6	1.4	2.5	8.5	8.7	13.0	33.2	24.3	13.9	13.8	16.4	29.4	18.4	14.9	66.4
Children 11-18	27.1	22.3	32.0	82.6	3.6	1.5	1.3	2.3	7.8	8.1	12.1	30.7	22.4	12.9	12.8	15.2	29.4	18.4	14.9	66.4
Men 19-64	27.1	22.3	32.0	82.6	3.3	1.4	1.3	2.1	7.3	7.5	11.3	28.7	21.0	12.0	11.9	14.2	29.4	18.4	14.9	66.4
Women 19-64	27.1	22.3	32.0	82.6	4.2	1.7	1.6	2.7	9.2	9.5	14.1	35.9	26.3	15.1	14.9	17.8	29.4	18.4	14.9	66.4
Adults 19-64	27.1	22.3	32.0	82.6	3.7	1.5	1.4	2.4	8.1	8.4	12.5	31.9	23.3	13.4	13.3	15.8	29.4	18.4	14.9	66.4
Men 65+	27.1	22.3	32.0	82.6	3.8	1.6	1.4	2.5	8.4	8.7	13.0	33.0	24.1	13.8	13.7	16.3	29.4	18.4	14.9	66.4
Women 65+	27.1	22.3	32.0	82.6	4.8	2.0	1.8	3.1	10.4	10.7	16.0	40.7	29.8	17.1	16.9	20.1	29.4	18.4	14.9	66.4
Adults 65+	27.1	22.3	32.0	82.6	4.2	1.8	1.6	2.7	9.3	9.6	14.3	36.4	26.7	15.3	15.2	18.0	29.4	18.4	14.9	66.4
Men 65-74	27.1	22.3	32.0	82.6	3.8	1.6	1.4	2.4	8.3	8.6	12.8	32.6	23.9	13.7	13.6	16.1	29.4	18.4	14.9	66.4
Women 65-74	27.1	22.3	32.0	82.6	4.7	1.9	1.7	3.0	10.2	10.5	15.7	40.0	29.2	16.7	16.6	19.8	29.4	18.4	14.9	66.4
Adults 65-74	27.1	22.3	32.0	82.6	4.2	1.7	1.6	2.7	9.2	9.5	14.1	35.9	26.3	15.1	14.9	17.8	29.4	18.4	14.9	66.4
Men 75+	27.1	22.3	32.0	82.6	3.9	1.6	1.5	2.5	8.5	8.8	13.1	33.3	24.4	14.0	13.9	16.5	29.4	18.4	14.9	66.4
Women 75+	27.1	22.3	32.0	82.6	4.8	2.0	1.8	3.1	10.6	10.9	16.3	41.5	30.4	17.4	17.3	20.5	29.4	18.4	14.9	66.4
Adults 75+	27.1	22.3	32.0	82.6	4.3	1.8	1.6	2.8	9.4	9.7	14.5	37.0	27.0	15.5	15.4	18.3	29.4	18.4	14.9	66.4

[1] RDIs from the Scientific Advisory Committee on Nutrition (SACN) [3]: long-chain n-3 PUFA, 200-450 mg/day; n-6, <10% EI; ALNA, >0.2% EI; LA, >1% EI; and EI for the different demographics as in SACN reference values [4]. [1] ALA; alpha-linolenic acid. [2] LA; alpha-linolenic acid. [3] EPA, eicosapentaenoic acid and DHA, docosahexaenoic acid.

Table S5. Predicted fatty acid intakes from beef produced in four different beef feed systems (Intensive, INT; Conventional, CON; Organic, ORG; Pasture-Based, PB) as a percentage of recommended daily intake (RDI)[1] across sex and age based on the EAT LANCET: Eat Lancet report [6] of 98 g beef/week (14 g/day); assuming average fat content of beef been 10.61 g/100g (averaged from 23 categories of cooked beef cuts), and fatty acid content of beef fat to be 93.5%, according to the McCance and Widdowson's composition of foods integrated dataset [2].

	SFA[2]				Trans FA[3]				MUFA[4]				cis-PUFA[5]			
Demographic group	INT	CON	ORG	PB	INT	CON	ORG	PB	INT	CON	ORG	PB	INT	CON	ORG	PB
Children 1.5-3					2.5	3.0	2.0	1.9	4.5	5.4	5.1	4.2		0.6	0.5	0.3
Boys 4-10	3.6	3.5	3.5	3.2	1.5	1.8	1.2	1.1	2.7	3.2	3.0	2.5		0.4	0.3	0.2
Girls 4-10	3.8	3.8	3.7	3.5	1.6	1.9	1.2	1.2	2.8	3.4	3.2	2.7		0.4	0.3	0.2
Children 4-10	3.7	3.7	3.6	3.3	1.5	1.9	1.2	1.2	2.7	3.3	3.1	2.6		0.4	0.3	0.2
Boys 11-18	2.2	2.2	2.2	2.0	0.9	1.1	0.7	0.7	1.7	2.0	1.9	1.5		0.2	0.2	0.1
Girls 11-18	2.6	2.6	2.5	2.3	1.1	1.3	0.8	0.8	1.9	2.3	2.2	1.8		0.3	0.2	0.1
Children 11-18	2.4	2.4	2.3	2.2	1.0	1.2	0.8	0.8	1.8	2.1	2.0	1.7		0.3	0.2	0.1
Men 19-64	2.2	2.2	2.2	2.0	0.9	1.1	0.7	0.7	1.7	2.0	1.9	1.6		0.2	0.2	0.1
Women 19-64	2.8	2.8	2.7	2.5	1.2	1.4	0.9	0.9	2.1	2.5	2.4	1.9		0.3	0.3	0.1
Adults 19-64	2.5	2.5	2.4	2.3	1.0	1.3	0.8	0.8	1.9	2.2	2.1	1.7		0.3	0.2	0.1
Men 65+	2.6	2.6	2.5	2.3	1.1	1.3	0.8	0.8	1.9	2.3	2.2	1.8		0.3	0.2	0.1
Women 65+	3.2	3.2	3.1	2.9	1.3	1.6	1.0	1.0	2.4	2.8	2.7	2.2		0.3	0.3	0.1
Adults 65+	2.9	2.8	2.8	2.6	1.2	1.4	0.9	0.9	2.1	2.5	2.4	2.0		0.3	0.3	0.1
Men 65-74	2.6	2.5	2.5	2.3	1.0	1.3	0.8	0.8	1.9	2.3	2.1	1.8		0.3	0.2	0.1
Women 65-74	3.1	3.1	3.0	2.8	1.3	1.6	1.0	1.0	2.3	2.8	2.6	2.2		0.3	0.3	0.1
Adults 65-74	2.8	2.8	2.7	2.5	1.2	1.4	0.9	0.9	2.1	2.5	2.4	1.9		0.3	0.3	0.1
Men 75+	2.6	2.6	2.5	2.4	1.1	1.3	0.9	0.8	1.9	2.3	2.2	1.8		0.3	0.2	0.1
Women 75+	3.3	3.2	3.2	2.9	1.3	1.6	1.1	1.0	2.4	2.9	2.7	2.3		0.3	0.3	0.2
Adults 75+	2.9	2.9	2.8	2.6	1.2	1.4	0.9	0.9	2.2	2.6	2.4	2.0		0.3	0.3	0.1

[1] RDIs from the Scientific Advisory Committee on Nutrition (SACN) [3]: SFA, <10% energy intake (EI); trans-fat, <2% EI; MUFA, 12 % EI; cis-PUFA, 6% EI; and EI for the different demographics as in SACN reference values [4]. [2] SFA, Saturated Fatty Acids. [3] Trans FA, Trans- Fatty Acids. [4] MUFA; Monounsaturated Fatty Acids. [5] cis-PUFA, cis-Polyunsaturated Fatty Acids.

Table S6. Predicted fatty acid intakes from beef produced in four different beef feed systems (Intensive, INT; Conventional, CON; Organic, ORG; Pasture-Based, PB) as a percentage of recommended daily intake (RDI)[1] across sex and age based on the EAT LANCET: Eat Lancet report [6] of 98 g beef/week (14 g/day); assuming average fat content of beef been 10.61 g/100g (averaged from 23 categories of cooked beef cuts), and fatty acid content of beef fat to be 93.5%, according to the McCance and Widdowson's composition of foods integrated dataset [2].

	Omega-3				Omega-6				ALA[1]				LA[2]				EPA+DHA[3]			
Demographic group	INT	CON	ORG	PB	INT	CON	ORG	PB	INT	CON	ORG	PB	INT	CON	ORG	PB	INT	CON	ORG	PB
Children 1.5-3	5.4	4.5	6.4	16.5	1.8	0.7	0.7	1.2	3.9	4.1	6.1	15.5	11.3	6.5	6.4	7.6	5.9	3.7	3.0	13.3
Boys 4-10	5.4	4.5	6.4	16.5	1.1	0.4	0.4	0.7	2.3	2.4	3.6	9.1	6.7	3.8	3.8	4.5	5.9	3.7	3.0	13.3
Girls 4-10	5.4	4.5	6.4	16.5	1.1	0.5	0.4	0.7	2.5	2.6	3.8	9.8	7.1	4.1	4.1	4.8	5.9	3.7	3.0	13.3
Children 4-10	5.4	4.5	6.4	16.5	1.1	0.5	0.4	0.7	2.4	2.5	3.7	9.4	6.9	4.0	3.9	4.7	5.9	3.7	3.0	13.3
Boys 11-18	5.4	4.5	6.4	16.5	0.7	0.3	0.2	0.4	1.5	1.5	2.2	5.7	4.2	2.4	2.4	2.8	5.9	3.7	3.0	13.3
Girls 11-18	5.4	4.5	6.4	16.5	0.8	0.3	0.3	0.5	1.7	1.7	2.6	6.6	4.9	2.8	2.8	3.3	5.9	3.7	3.0	13.3
Children 11-18	5.4	4.5	6.4	16.5	0.7	0.3	0.3	0.5	1.6	1.6	2.4	6.1	4.5	2.6	2.6	3.0	5.9	3.7	3.0	13.3
Men 19-64	5.4	4.5	6.4	16.5	0.7	0.3	0.3	0.4	1.5	1.5	2.3	5.7	4.2	2.4	2.4	2.8	5.9	3.7	3.0	13.3
Women 19-64	5.4	4.5	6.4	16.5	0.8	0.3	0.3	0.5	1.8	1.9	2.8	7.2	5.3	3.0	3.0	3.6	5.9	3.7	3.0	13.3
Adults 19-64	5.4	4.5	6.4	16.5	0.7	0.3	0.3	0.5	1.6	1.7	2.5	6.4	4.7	2.7	2.7	3.2	5.9	3.7	3.0	13.3
Men 65+	5.4	4.5	6.4	16.5	0.8	0.3	0.3	0.5	1.7	1.7	2.6	6.6	4.8	2.8	2.7	3.3	5.9	3.7	3.0	13.3
Women 65+	5.4	4.5	6.4	16.5	1.0	0.4	0.4	0.6	2.1	2.1	3.2	8.1	6.0	3.4	3.4	4.0	5.9	3.7	3.0	13.3
Adults 65+	5.4	4.5	6.4	16.5	0.8	0.4	0.3	0.5	1.9	1.9	2.9	7.3	5.3	3.1	3.0	3.6	5.9	3.7	3.0	13.3
Men 65-74	5.4	4.5	6.4	16.5	0.8	0.3	0.3	0.5	1.7	1.7	2.6	6.5	4.8	2.7	2.7	3.2	5.9	3.7	3.0	13.3
Women 65-74	5.4	4.5	6.4	16.5	0.9	0.4	0.3	0.6	2.0	2.1	3.1	8.0	5.8	3.3	3.3	4.0	5.9	3.7	3.0	13.3
Adults 65-74	5.4	4.5	6.4	16.5	0.8	0.3	0.3	0.5	1.8	1.9	2.8	7.2	5.3	3.0	3.0	3.6	5.9	3.7	3.0	13.3
Men 75+	5.4	4.5	6.4	16.5	0.8	0.3	0.3	0.5	1.7	1.8	2.6	6.7	4.9	2.8	2.8	3.3	5.9	3.7	3.0	13.3
Women 75+	5.4	4.5	6.4	16.5	1.0	0.4	0.4	0.6	2.1	2.2	3.3	8.3	6.1	3.5	3.5	4.1	5.9	3.7	3.0	13.3
Adults 75+	5.4	4.5	6.4	16.5	0.9	0.4	0.3	0.6	1.9	1.9	2.9	7.4	5.4	3.1	3.1	3.7	5.9	3.7	3.0	13.3

[1] RDIs from the Scientific Advisory Committee on Nutrition (SACN) [3]: long-chain n-3 PUFA, 200-450 mg/day; n-6, <10% EI; ALNA, >0.2% EI; LA, >1% EI; and EI for the different demographics as in SACN reference values [4]. [1] ALA; alpha-linolenic acid. [2] LA; alpha-linolenic acid. [3] EPA, eicosapentaenoic acid and DHA, docosahexaenoic acid.

References

1. Bates, B.; Collins, D.; Jones, K.; Page, P.; Roberts, C.; Swan, G. National Diet and Nutrition Survey Rolling programme Years 9 to 11 (2016/2017 to 2018/2019). Public Health England. Available from: https://www.gov.uk/government/statistics/ndns-results-from-years-9-to-11-2016-to-2017-and-2018-to-2019 (accessed July 2021) 2020.
2. McCance, R.A.; Widdowson, E.M. McCance and Widdowson's composition of foods integrated dataset. 2015.
3. Scientific Advisory Committee on Nutrition. Saturated fats and health; Scientific Advisory Committee on Nutrition: London, 2019.
4. Nutrition, S.A.C.o. Dietary reference values for energy; The Stationery Office: 2012.
5. NHS. Red meat and the risk of bowel cancer. Available online: https://www.nhs.uk/live-well/eat-well/red-meat-and-the-risk-of-bowel-cancer (accessed on 01/12/2021).
6. Willett, W.; Rockström, J.; Loken, B.; Springmann, M.; Lang, T.; Vermeulen, S.; Garnett, T.; Tilman, D.; DeClerck, F.; Wood, A.; et al. Food in the Anthropocene: the EAT–Lancet Commission on healthy diets from sustainable food systems. The Lancet (British edition) 2019, 393, 447-492, doi:10.1016/S0140-6736(18)31788-4.
